# Hemispheric efficiency during the day: the influence of circadian preference and time-of-day on verbal and visuospatial tasks

**DOI:** 10.3389/fpsyg.2026.1885699

**Published:** 2026-09-03

**Authors:** Federica Giudetti, Lorenzo Tonetti, Vincenzo Natale, Marco Fabbri

**Affiliations:** Department of Psychology “Renzo Canestrari,” University of Bologna, Bologna, Italy

**Keywords:** chronotype, divided visual field, hemispheric activation, landmark task, semantic categorization task, time-of-day effect

## Abstract

**Introduction:**

Cognitive performance fluctuates daily due to the interaction between circadian and homeostatic processes, which may differently activate the two hemispheres. This study investigated the effects of time-of-day, circadian typology, and the synchrony effect on semantic categorization and visuospatial processing tasks with potential hemispheric lateralization.

**Methods:**

A sample of 113 healthy young women (20.60 ± 2.60 years) performed two divided visual field tasks (semantic categorization and vertical landmark) in morning (08:00) and early afternoon (13:00) sessions.

**Results:**

In the semantic task, an interaction between circadian preference and spatial position showed a progressive shift toward a right-visual-field preference from morning- to evening-types. A triple interaction (typology × word dominance × response type) revealed that for yes- responses, extreme chronotypes were faster with high-dominance items, while intermediate-types were faster with low-dominance ones. For no-responses, intermediate and evening-types favored low-dominance items, whereas morning-types showed no difference. In the landmark task, a Time-of-Day × Position interaction showed a right-visual-field advantage in the morning, which shifted to a left-visual-field advantage in the afternoon.

**Discussion:**

Overall, findings indirectly suggest daily hemispheric variations based on task demands, with the left hemisphere more active in the morning and the right in the afternoon, and a modulation of circadian preference, at least for semantic processing.

## 1 Introduction

It is commonly documented that cognitive performance and mood vary predictably across the 24-h day (Carrier and Monk, 2000; Czeisler and Gooley, 2007; Schmidt et al., 2007). These rhythms are linked to the sleep–wake cycle and are driven by the internal circadian clock, a biological pacemaker with a period of approximately 1 day (circa diem). The sleep–wake cycle is regulated by two interacting processes: the homeostatic sleep pressure (Process S) and the circadian pacemaker (Process C) (Borbély, 1982; Borbély et al., 2016; Rogers et al., 2003). Within the two-process model, Process S is defined as a homeostatic, sleep-promoting mechanism that continuously accumulates during wakefulness. This accumulation is associated with a decline in cognitive performance and alertness, alongside an increase in subjective sleepiness (Borbély, 1982; Borbély et al., 2016). In contrast, Process C refers to a near-24-h endogenous oscillatory process, characterized by its highest levels in the early evening and the lowest in the early morning hours (Van Dongen and Dinges, 2003). This clock-like mechanism is synchronized with environmental time cues under normal conditions, is governed by the suprachiasmatic nuclei in the anterior hypothalamus and can be indirectly assessed through measures such as core body temperature rhythms (Schmidt et al., 2007).

From a cognitive perspective, the two-process model explains daily fluctuations in neurobehavioral efficiency as arising from increasing homeostatic sleep pressure, circadian modulation of optimal performance, or their interaction (Carrier and Monk, 2000; Czeisler and Gooley, 2007). The interaction between Process S and Process C is particularly evident in alertness (Natale and Cicogna, 1996; Van Dongen and Dinges, 2005), a behavioral rather than purely physiological construct (Adan et al., 2012). Cognitive performance is therefore more efficient when testing occurs in synchrony with an individual’s peak alertness. This phenomenon, known as the synchrony effect (Chauhan et al., 2025; May and Hasher, 1998; May et al., 2023; Wiłkość-Dêbczyńska and Liberacka-Dwojak, 2023), is closely related to individual differences in circadian preference (Adan et al., 2012; Ceglarek et al., 2021; May et al., 2023; Natale et al., 2003). It is typically distributed along a continuum approximating a Gaussian curve, with intermediate types clustering near the center and showing no strong temporal preference (Natale et al., 2003; Palmero et al., 2024). At the extremes of this distribution are morning-types, who prefer morning activities and exhibit peak arousal early in the day, and evening-types, who prefer evening activities and reach peak arousal later in the day (Palmero et al., 2024; Schmidt et al., 2007). Accordingly, the synchrony effect predicts that morning-types perform better in the morning, whereas evening-types show superior performance in the afternoon or evening across various cognitive tasks (Blatter and Cajochen, 2007; Ceglarek et al., 2021; May et al., 2023; Schmidt et al., 2007; Wiłkość-Dêbczyńska and Liberacka-Dwojak, 2023). Overall, the literature indicates that time of day influences performance across multiple cognitive domains. For instance, evening sessions are associated with poorer retrieval efficiency in semantic classification (Fabbri et al., 2013) but higher visuospatial attention (Fimm et al., 2016). These effects are further modulated by interindividual differences in circadian preference (Blatter and Cajochen, 2007; Ceglarek et al., 2021; May et al., 2023; Schmidt et al., 2007; Wiłkość-Dêbczyńska and Liberacka-Dwojak, 2023), particularly regarding long-term memory (Anderson et al., 1991; Petros et al., 1990) and attention (Fabbri et al., 2017; Natale et al., 2003; Palmero et al., 2024; Van Opstal et al., 2022). Conversely, in their systematic review, Chauhan et al. (2025; see also Adan et al., 2012) reported that over 70% of reviewed studies on young adults found no time-of-day effect, more than 80% reported no main effect of chronotype, and only about 45% demonstrated a synchrony effect. Authors highlighted several methodological issues to explain these inconsistencies. These include variability in testing schedules to accurately capture chronotype or synchrony effects (e.g., Hidalgo et al., 2004), discrepancies in procedures and cognitive parameters (Chauhan et al., 2025), a limited range of chronotype scores that may have attenuated the synchrony effect (e.g., May et al., 2023), and sex-related differences in cognitive performance (e.g., Killgore and Killgore, 2007).

One factor that may account for these inconsistencies is hemispheric involvement during task performance. Natale and colleagues, using actigraphy, demonstrated distinct circadian phase relationships between the two hemispheres (Natale, 2002; Natale et al., 2007, 2022). Specifically, Natale (2002), in a sample of right-handed participants, reported greater motor activity in the non-dominant hand (i.e., right hemisphere) compared to the dominant hand (i.e., left hemisphere) during the first 2 h of sleep and in the 4 h preceding sleep onset. This pattern may reflect a phase difference between hemispheres, with the right hand reaching its acrophase slightly but significantly earlier than the left (by approximately 12 min). This finding was partially replicated in a study of 51 right-handed individuals monitored via actigraphy across three consecutive days (baseline, sleep deprivation, and recovery), which showed higher motor activity in the left hand (right hemisphere) relative to the right hand (left hemisphere) prior to sleep onset under baseline conditions (Natale et al., 2007). More recently, Natale et al. (2022) investigated how chronotype and dominant/non-dominant hemisphere influence motor activity patterns in 113 healthy, right-handed participants categorized as morning-, intermediate-, or evening-types. Their study yielded two key findings. First, a shift in acrophase was observed between the dominant and non-dominant hands; specifically, non-dominant hand (right hemisphere) activity showed superiority late in the evening, irrespective of the participant’s chronotype. This finding supports the existence of interhemispheric phase differences, suggesting that the relative dominance of non-dominant hand movements may stem from distinct circadian phases within each hemisphere. Consequently, authors proposed that the left hemisphere might be more sensitive to Process S, leading to earlier deactivation, whereas the right hemisphere may be more strongly influenced by Process C, thereby maintaining activity later into the evening despite accumulated sleep pressure. Second, morning- and evening-types differed in their motor activity patterns at bedtime and wake-up times (± 4 h before and after bedtime or wake-up times): evening-types showed greater left-hand activity than that reported in the right-hand before sleep, whereas morning-types exhibited greater right-hand activity than that observed in the left-hand after awakening. Within the framework of the two-process model, morning-types may be more sensitive to left-hemisphere functioning, which is primarily related to homeostatic regulation (Process S), whereas evening-types may be more influenced by right-hemisphere activity, which is more closely linked to circadian regulation (Process C), potentially explaining their delayed sleep timing (see also Mongrain et al., 2011; Schmidt et al., 2012). In line with this assumption, it has also been observed that extreme chronotypes differ in the kinetics of the homeostatic process (e.g., Taillard et al., 2003) and circadian process (e.g., Putilov and Donskaya, 2022), after controlling for age differences. These findings indicate not only differences between chronotypes for the two-process model, but also for hemispheric functioning and processing.

Differences in hemispheric preference across chronotypes may also manifest in task performance. Morning-types have been associated with a preference for left-hemisphere processing, whereas evening-types show a relative preference for right-hemisphere processing (Díaz-Morales and Escribano, 2013; Fabbri et al., 2007). This distinction may underlie diurnal variations in cognitive performance, with verbal tasks (left hemisphere) showing better performance in the morning and spatial tasks (right hemisphere) showing improved performance in the evening (Corbera and Grau, 1993; Corbera et al., 1993; Natale et al., 2003). Consistent with this view, Corbera and Grau (1993), in a sample of 48 right-handed women tested at four times of day (09:30, 12:00, 16:00, and 19:45), reported both time-of-day and chronotype effects. Specifically, they observed a left-hemisphere advantage at midday and a right-hemisphere advantage in the evening, as well as more errors in the left visual field for morning-types during verbal tasks and in the right visual field for evening-types during spatial tasks. Using a semantic categorization task (Tilley and Warren, 1983) administered at three times of day (09:00–10:00, 13:00–14:00, and 17:00–18:00), Fabbri et al. (2013) found both time-of-day and chronotype effects. Participants showed faster responses to high-dominance (central) category items compared to low-dominance (peripheral) items, particularly in morning and afternoon sessions. Moreover, morning-types exhibited a larger performance difference between high- and low-dominance items, whereas this difference was reduced in evening-types. According to models of hemispheric asymmetry in semantic processing, where representations are “fine” or “focused” in the left hemisphere and “coarse” or “diffuse” in the right (Beeman, 1998; Chiarello, 1988), these findings suggest that morning-types rely more on focused semantic activation, while evening-types engage broader, more diffuse semantic networks (Deacon et al., 2004). However, two studies challenge these previous findings. First, Killgore and Killgore (2007) identified an association between eveningness and higher verbal Intelligence Quotients (IQs), a correlation that proved stronger when analyzing females exclusively. Although authors used a psychometrically validated tool, the study was cross-sectional. Second, Palmero et al. (2024) evaluated morning- and evening-types at 08:00 and 20:30 using a category semantic priming task. They found that while morning-types maintained consistent performance across sessions (i.e., indicating a proactive control style), evening-types were significantly affected by the time of day, exhibiting a shift from automatic to controlled processing (i.e., indicating a reactive control style). Notably, these latter findings were only marginally significant and were not systematically observed across both accuracy and response times. Therefore, focusing on the semantic categorization (Chiarello, 1988; Deacon et al., 2004), by testing an all-female sample, in a sort of replication of the study by Corbera and Grau (1993), through a divided visual field paradigm could further clarify how hemispheric processing and chronotype jointly modulate semantic categorization (Corbera and Grau, 1993; Fabbri et al., 2007, 2013).

Similar patterns have been observed in visuospatial tasks. For instance, Monk and Leng (1986) reported peak performance in a serial search task in the late afternoon (17:00), consistent with findings in controlled visuospatial processing tasks (van der Heijden et al., 2010), although some studies report different results (Natale et al., 2003; Song and Stough, 2000). Fimm et al. (2016) assessed time-of-day variations in visuospatial attention across five sessions (09:00, 12:00, 15:00, 18:00, and 21:00). In a sample of 15 healthy participants, authors measured body temperature alongside covert and overt orienting of attention using both unilateral and bilateral stimulus presentations. The results demonstrated that both covert and overt orienting improved throughout the day, showing no asymmetries between the left and right visual hemifields. However, a significant association with body temperature was observed exclusively for covert orienting. These findings support the notion that right-hemisphere activation may be particularly sensitive to circadian influences (Process C), although different visuospatial tasks, methodological procedures, and time-of-day were used. Regarding chronotype differences, evening-types have been shown to outperform morning-types in visuospatial working memory tasks (e.g., forward Corsi block-tapping test; Bettencourt et al., 2025) and mental rotation tasks (Nishida et al., 2022), with a stronger synchrony effect observed in evening-types. In a spatial attention task (landmark task), Dorrian et al. (2017) found that attentional biases varied with both chronotype and time of testing (06:00 and 23:00): non-optimal testing times were associated with a rightward attentional shift, whereas optimal times produced a leftward shift, particularly in morning-types (see also Manly et al., 2005). A deep inspection of their Figure 1 (page 404) revealed that evening-types basically reported a general pseudoneglect (i.e., overestimation of the left side of the line; Jewell and McCourt, 2000) in both testing sessions, which could reflect a greater right hemisphere activation during spatial tasks (Çiçek et al., 2009), suggesting at least a preference of evening-types for the right hemisphere. However, this study has methodological limitations, given that the authors used only the last question of the reduced Morningness-Eveningness Questionnaire or rMEQ (Adan and Almirall, 1991) for the assessment of extreme chronotypes, casting doubts for the identification of morning- and evening-types, used a line bisection task for the visuospatial assessment, casting doubts for the investigation of hemispheric asymmetries, and selected extreme session times not in line with those reported in other studies. Indeed, the complete administration of the rMEQ allows a validated identification of chronotypes, the vertical version of the landmark task has been shown to preferentially engage right parietal regions more than other visuospatial tasks (Seydell-Greenwald et al., 2017, 2019), and the use of other times-of-day is more suitable for investigating hemispheric asymmetries. Moreover, the adoption of a divided visual field paradigm could be useful to target the involvement of both hemispheres in visuospatial processing.

**FIGURE 1:**
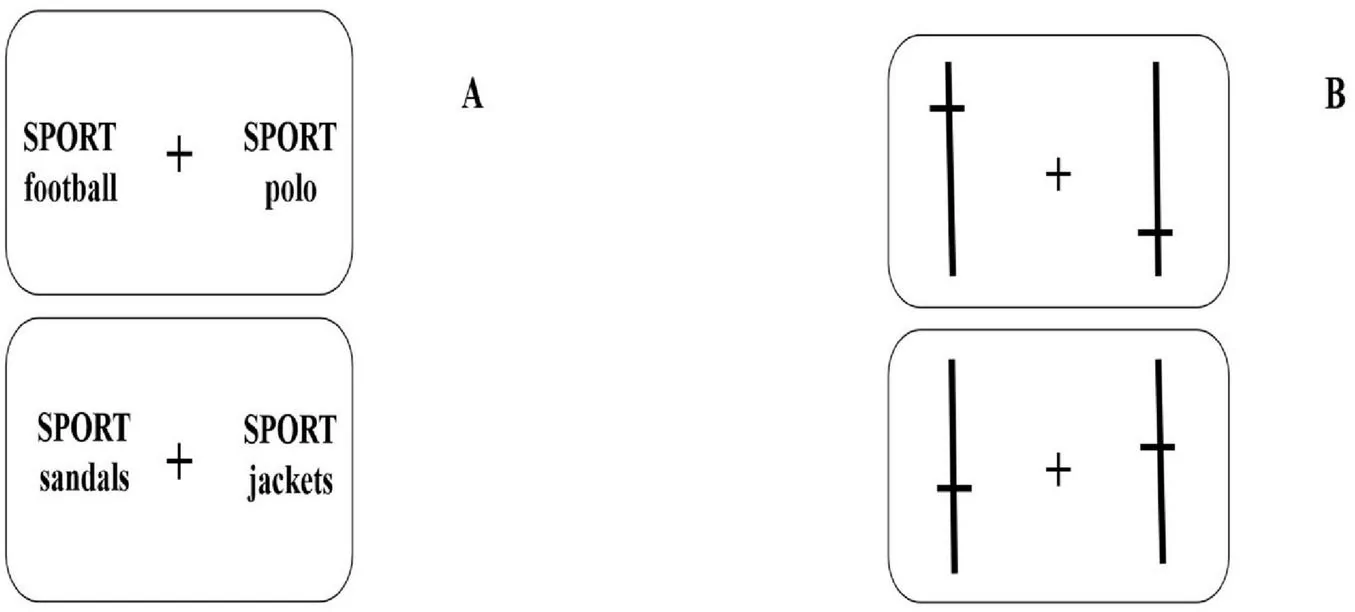
**(A)** Example trials from the semantic categorization task, illustrating category-word pairs requiring yes-responses for high- and low-dominance members (upper panel) and no-responses for low- and high-dominance members (lower panel). **(B)** Example trials from the landmark task, showing easy trials (upper panel) and hard trials (lower panel), with the longer segment located either above or below the bisection mark. In all cases, stimuli were presented either to the left or right of a central fixation point within a divided visual field paradigm (see Design and procedure for details).

The present study aimed to investigate the effects of time of day, circadian typology, and their interaction (i.e., the synchrony effect) on semantic categorization and visuospatial processing. To this end, we employed both a semantic categorization task (Fabbri et al., 2013) and a vertical landmark task (Seydell-Greenwald et al., 2019), combined with a divided visual field paradigm. Both tasks were administered in the morning (08:00) and afternoon (13:00) to allow for a direct comparison between the current data and existing literature. Regarding the semantic categorization task, Fabbri et al. (2013) utilized morning, afternoon, and evening testing sessions, following a methodology similar to that of Corbera and Grau (1993), selecting the main testing sessions to detect differences between hemispheres and chronotypes. For the visuospatial task, Monk and Leng (1986) and Natale et al. (2003) employed six testing intervals (08:00, 11:00, 14:00, 17:00, 20:00, and 23:00; see also Nishida et al., 2022, who used 08:00 and 20:00). Meanwhile, van der Heijden et al. (2010) tested participants in the early morning (08:30), late morning (10:00), and afternoon (13:00), aligning with the approaches of Song and Stough (2000) as well as Bettencourt et al. (2025), who used morning (09:00) and afternoon (15:00) sessions. Consequently, our selection of testing times is well-suited to evaluate variations in hemispheric lateralization while ensuring comparability with previous findings. For the semantic task, we expected to replicate previous findings (Fabbri et al., 2013), with faster detection of high-dominance category members in the morning and a larger difference between high- and low-dominance items in morning-types, particularly for right visual field stimuli (left hemisphere processing). For the landmark task, we expected faster responses in the afternoon, especially for stimuli presented in the left visual field (right hemisphere processing), and superior performance in evening-types. In both tasks, the synchrony effect was expected to manifest as improved performance when testing time aligned with individual circadian preference.

## 2 Materials and methods

### 2.1 Participants

The study initially recruited 138 female participants, with exclusion criteria related to neurological or physical conditions, as well as not being native Italian speakers. Additionally, participants whose overall accuracy fell below 80% in at least one experimental session were excluded from the analyses (*n* = 25). The final sample therefore consisted of 113 participants (mean age = 20.60 years, SD = 2.60 years). Although no a priori power analysis was conducted, our sample size did not compromise statistical power. For the semantic categorization task, our sample provided a balanced middle ground between the 170 participants in Fabbri et al. (2013) and the 48 participants in Corbera and Grau (1993). Concurrently, for the visuospatial task, our sample size was larger than those reported in previous studies, which ranged from 15 participants (Fimm et al., 2016) to 530 (Dorrian et al., 2017).

The sample was restricted to female participants to reduce variability associated with well-documented sex differences in cognitive performance in semantic categorization (e.g., Pasterski et al., 2011), line bisection (e.g., Hausmann et al., 2002), and hemispheric specialization (e.g., Gur and Gur, 2017). In addition, we adopted the same methodological procedure adopted by Corbera and Grau (1993). This choice was intended to minimize potential confounding effects and more clearly isolate time-of-day and influences related to morningness-eveningness preference. Furthermore, the sample could not be affected by gender differences in chronotype distribution (e.g., Tonetti et al., 2008).

Handedness was assessed using the Edinburgh Handedness Inventory (EHI; Oldfield, 1971). The mean EHI score was 73.62 (SD = 44.59), with 5 left-handed participants (*M* = −82.60; SD = 23.15), 9 ambidextrous participants (*M* = −1.54; SD = 29.86), and 99 right-handed participants (*M* = 88.34; SD = 16.76).

### 2.2 Materials

#### 2.2.1 Alertness

Core body temperature was assessed using an axillary digital thermometer (°C) as an indirect index of objective alertness, following previous research (Correa et al., 2017; Natale et al., 2003). Measurement in the right axilla provides a non-invasive and cost-effective proxy of alertness (Wright et al., 2002).

Subjective alertness and affect were assessed using the Global Vigor and Affect scale (GVA; Monk, 1989), an 8-item visual analog scale. For each item, participants marked their response on a 10-cm line anchored by “not at all” and “very much.” Four items (tenseness, happiness, sadness, calmness) measured affect, whereas the remaining four items (vigor, tiredness, effort required for daily tasks, sleepiness) measured subjective vigor (i.e., subjective alertness). Higher scores indicate better mood and greater alertness.

#### 2.2.2 Morningness-eveningness preferences

Circadian preference was assessed using the reduced Morningness–Eveningness Questionnaire (rMEQ; Adan and Almirall, 1991), in its Italian version (Natale, 1999). The rMEQ consists of five items derived from the original Morningness–Eveningness Questionnaire (Horne and Östberg, 1976). Total scores range from 4 (extreme eveningness) to 25 (extreme morningness). Italian cut-off scores were used to classify participants into evening-types (≤ 11), intermediate-types, and morning-types (≥ 18).

#### 2.2.3 Cognitive tasks

Tasks were administered on a desktop computer using E-Prime 2.0 software (Schneider et al., 2012), with participants seated approximately 60 cm from a monitor (600 × 800 resolution) and responding via a standard QWERTY keyboard.

For the semantic categorization task, the paradigm used by Fabbri et al. (2013) was adapted. Each trial presented a category (uppercase) and a word (lowercase) simultaneously (e.g., VEHICLE–car) in Microsoft Sans Serif, 24-point font. Participants judged whether the word belonged to the category by pressing one of two keys (“l” or “d”), using the right and left index fingers, respectively. The mapping of yes/no responses to keys was counterbalanced across participants. Ten semantic categories were used (clothing, tool, fruit, toy, sport, vehicle, vegetables, weapons, furnishing). Half of the trials required a “yes” response (e.g., SPORT—football), and half required a “no” response (e.g., FRUIT—car). Additionally, 50% of trials involved high-dominance exemplars (central category members), and 50% involved low-dominance exemplars (peripheral members), for both yes and no responses. After 8 practice trials, the participants completed 80 experimental trials (Figure 1A).

For the landmark task, participants indicated whether the longer segment of a vertically bisected line was located above or below the bisection mark by pressing one of two keys (“l” or “d”), with response mapping counterbalanced. Vertical lines measured either 20 cm (650 pixels) or 12 cm (390 pixels) and were presented in Courier New, 18-point font. These lengths were selected based on prior evidence showing comparable judgment patterns, allowing data to be collapsed across lengths (Heber et al., 2010). Lines were bisected at 20 and 80% of their length (easy trials) or at 40 and 60% (hard trials). Lines bisected at 20 and 40% produced longer segments below the bisection mark, whereas those bisected at 60 and 80% produced longer segments above. After 8 practice trials, the participants completed 80 experimental trials (20 per condition; Figure 1B).

### 2.3 Design and procedure

Participants attended two laboratory sessions on consecutive days: one in the morning (08:00) and one in the afternoon (13:00). In each session, they completed both cognitive tasks, the GVA, and body temperature measurements. The rMEQ was administered only during the first session. The session order was counterbalanced: 52.21% of participants completed the afternoon session first (PM–AM), whereas 47.79% completed the morning session first (AM–PM). Each session lasted approximately 45 min and was conducted under controlled environmental conditions (constant lighting and temperature). At the beginning of each session, participants measured their body temperature, followed by completion of the GVA. As potential sources of variability, participants were instructed to maintain their regular sleep-wake and dietary habits on the days of testing, in order to maintain an ecological perspective. To avoid acute confounding effects on cognitive performance, they were requested to refrain from eating or consuming caffeinated beverages for at least 1 h before the start of each testing session (Adan et al., 2008; Mahoney et al., 2005). Thus, participants self-reported their number of hours (hh:mm) slept the night before [06:58 ± 01:33 and 07:05 ± 01:29 for first and second session, respectively, *t*(112) = −061, *p* = 0.55, *Cohen’s d* = 0.08] as well as whether (or not) they consumed caffeine and ate food, at least 1 h before the experimental session (caffeine intake: 31.90 and 35.40% of participants for first and second session, respectively; food intake: 85.00 and 73.50% of participants for first and second session, respectively). They then performed the cognitive tasks. A chin and forehead rest was used to maintain a fixed viewing distance (60 cm) and stabilize the head position. Participants were explicitly and repeatedly instructed to maintain strict central fixation on the central cross throughout each trial. For cognitive tasks, participants received written instructions emphasizing both speed and accuracy. A practice phase with feedback preceded the experimental trials; no feedback was provided thereafter. During this training phase for each task, the researchers visually monitored participants to ensure compliance with the setup. Each trial began with a central fixation cross presented for 500 ms, followed by stimulus presentation at either 25% (left visual field) or 75% (right visual field) of the screen width. Stimuli remained on screen until response. A fixation cross then reappeared for 1,000 ms before the next trial. Each task consisted of 80 randomized trials, with stimuli equally distributed across visual fields. Task order and response mappings were counterbalanced across participants and sessions. Each session included both tasks and lasted approximately 20 min for task completion. The same procedure was repeated in the second session. No dietary or sleep restrictions were imposed prior to testing.

A mixed design was used, with Time of Day (morning vs. afternoon) and Spatial Visual Field (left vs. right) as a within-subjects factor and Circadian Preference (morning-, intermediate-, evening-type) as a between-subjects factor. The study was approved by the Bioethics Committee of the University of Bologna (protocol number 0124290 of 23/04/2025) and was conducted in accordance with the Declaration of Helsinki. Written informed consent was obtained from all participants prior to enrolment. This study was not preregistered.

### 2.4 Data analysis

For both accuracy and reaction times (RTs), correct trials exceeding ± 3 standard deviations from the mean were excluded (approximately 2% in both tasks). Although only participants with ≥ 80% accuracy were included, we analyzed both RTs and accuracy, defined as the number of corrected trials.

Mean body temperature and subjective alertness (GVA) were analyzed using mixed ANOVAs with Circadian Preference as a between-subjects factor and Time of Day as a within-subjects factor.

For the semantic categorization task, RTs and accuracy were analyzed using a mixed ANCOVA with Circadian Preference as a between-subjects factor and Time of Day, Word Dominance (high vs. low membership), Stimulus Position (left- vs. right-visual field), and Response Type (yes vs. no) as within-subjects factors. Subjective alertness (difference in vigor between afternoon and morning sessions) was included as a covariate. For the landmark task, RTs and accuracy were analyzed using a mixed ANCOVA with Circadian Preference as a between-subjects factor and Time of Day, Task Difficulty (easy vs. hard), and Stimulus Position as within-subjects factors, again controlling for the difference in subjective vigor between sessions.

## 3 Results

According to the rMEQ cut-off scores, the sample comprised 20 evening-types, 67 intermediate-types, and 26 morning-types. No significant differences between chronotypes were observed for age [*F*(2, 110) = 2.76, *p* = 0.07, η^2^*_*p*_* = 0.05] or EHI score [*F*(2, 110) = 2.24, *p* = 0.11, η^2^*_*p*_* = 0.04]. Specifically, mean age was 20.96 years (SD = 2.84 years) for morning-types, 20.16 years (SD = 1.78 years) for intermediate-types, and 21.60 years (SD = 4.03 years) for evening-types. Mean EHI scores were 57.67 (SD = 61.99), 78.98 (SD = 39.99), and 76.39 (SD = 25.50) for morning-, intermediate-, and evening-types, respectively.

### 3.1 Objective alertness: body temperature

The 2 × 3 mixed ANOVA on mean body temperature revealed neither a significant main effect of Time of Day [*F*(1, 110) = 0.05, *p* = 0.83, η^2^*_*p*_* = 0.0001] nor a significant effect of Circadian Preference [*F*(2, 110) = 1.42, *p* = 0.25, η^2^*_*p*_* = 0.03]. The interaction between Time of Day and Circadian Preference was also not significant [*F*(2, 110) = 2.70, *p* = 0.07, η^2^*_*p*_* = 0.05]. The mean body temperature (and relative SD) for both testing sessions and each circadian preference is presented in Supplementary Table 1.

### 3.2 Subjective alertness: vigor score

**FIGURE 2:**
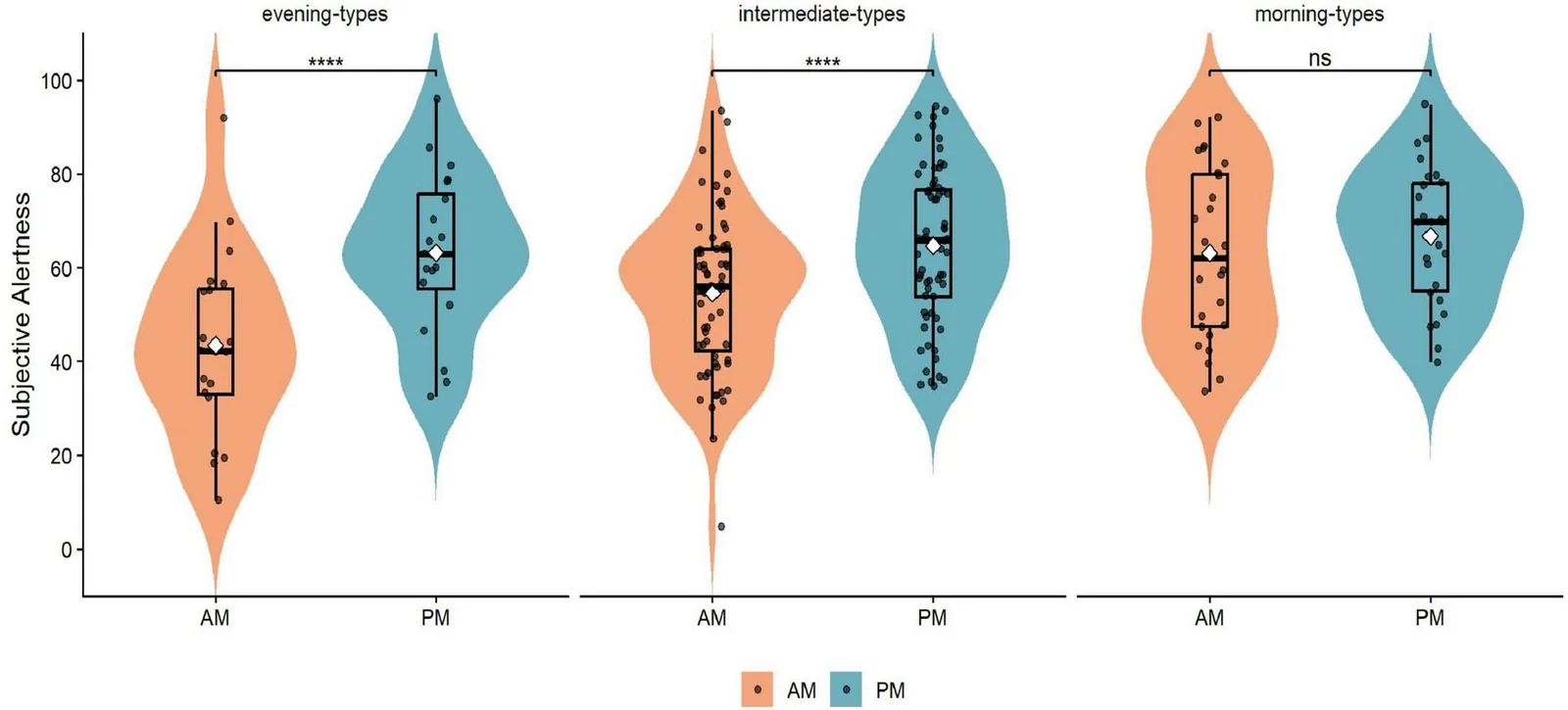
Distribution of subjective vigor scores during the morning (AM) and afternoon (PM) sessions across circadian typologies. Violin plots represent kernel density estimates; dots indicate individual observations, boxes represent the interquartile range, horizontal lines indicate medians, and white diamonds indicate means. Subjective vigor increased significantly from morning to afternoon in evening- and intermediate-types, but not in morning-types. ****p < 0.0001; ns, is not significant.

In Supplementary Table 1, the mean GVA vigor score (and relative SD) in both testing sessions and for morning-, intermediate, and evening-types is reported. The 2 × 3 mixed ANOVA on mean vigor scores (GVA) revealed a significant main effect of Time of Day [*F*(1, 110) = 23.49, *p* = 0.0001, η^2^*_*p*_* = 0.18], with participants reporting higher levels of subjective vigor in the afternoon (*M* = 64.87, SD = 16.02) compared to the morning (*M* = 53.72, SD = 18.23). A significant main effect of Circadian Preference was also observed [*F*(2, 110) = 4.46, *p* = 0.014, η^2^*_*p*_* = 0.08], indicating that morning-types (*M* = 64.93, SD = 16.57) reported higher vigor levels than evening-types (*M* = 53.34, SD = 18.24), with *p* = 0.011, but not (*p* = 0.19) with respect to intermediate-types (*M* = 59.62, SD = 16.58). Furthermore, the interaction between Time of Day and Circadian Preference was significant [*F*(2, 110) = 3.21, *p* = 0.04, η^2^*_*p*_* = 0.06], as shown in Figure 2. Tukey’s *post-hoc* test for unequal sample sizes indicated that evening-types reported markedly lower vigor in the morning (*M* = 43.48; SD = 19.58), followed by a substantial increase in the afternoon (*M* = 63.19; SD = 16.89), with *p* = 0.00001. Similarly, intermediate-types exhibited a moderate increase from morning (*M* = 54.54 ± 16.70) to afternoon (*M* = 64.70 ± 16.46), with *p* = 0.00001. In contrast, morning-types did not show a significant difference between morning (*M* = 63.14; SD = 18.42) and afternoon vigor (*M* = 66.71; SD = 14.72), with *p* = 0.37. Based on these findings, the difference between afternoon and morning subjective alertness was included as a covariate in subsequent analyses.

### 3.3 Semantic categorization task

The significant effects of the 3 × 2 × 2 × 2 × 2 mixed-design ANCOVA on mean RTs and accuracy for the semantic categorization task are reported below, whereas the complete statistical results are presented in Supplementary Table 3. In addition, Supplementary Table 2 reports mean RTs and numbers of corrected trials (and relative SD) of every circadian preference judging high- and low-dominance items presented at left- and right-visual field for yes- and no-responses in both testing sessions.

**FIGURE 3:**
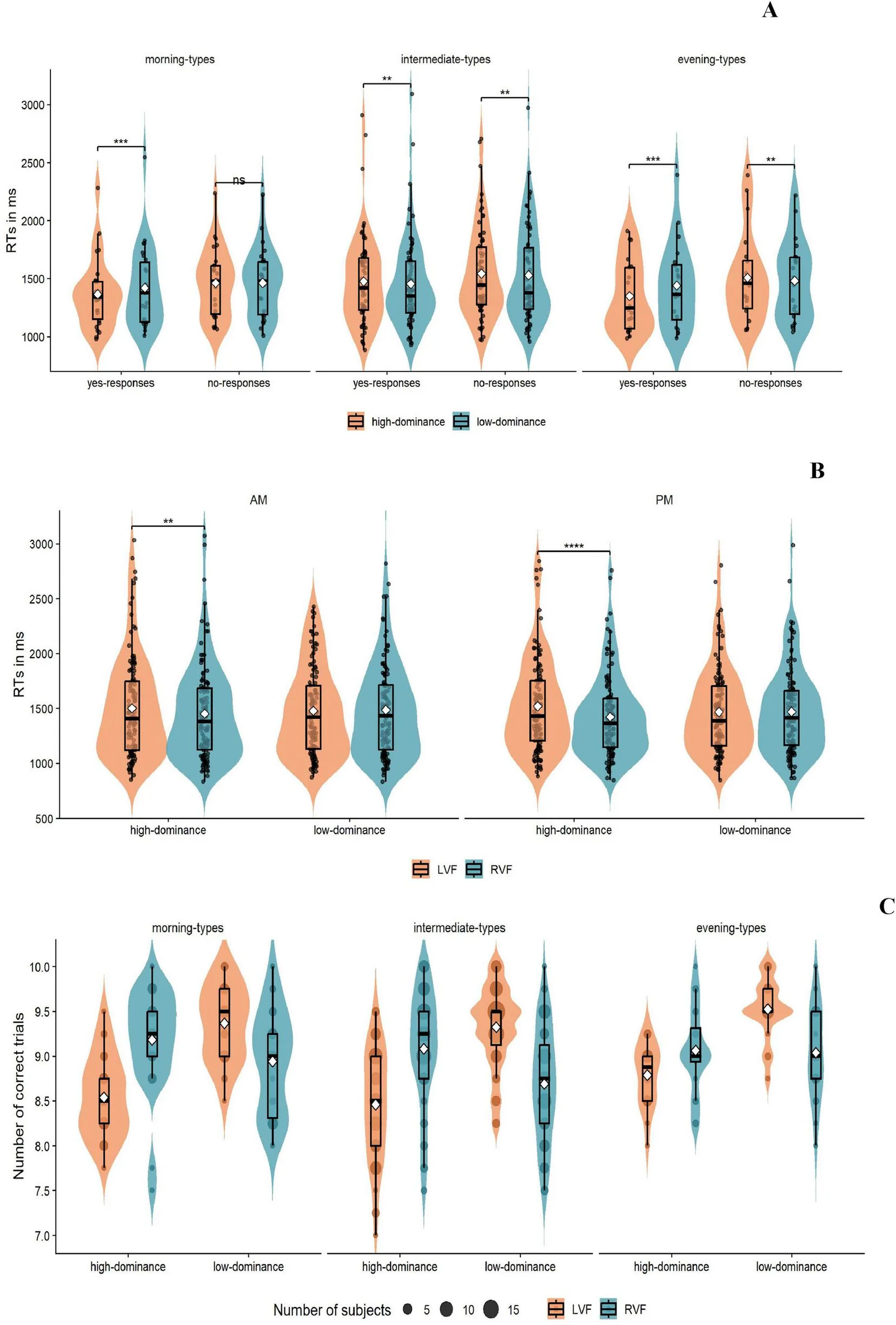
Violin plots represent kernel density estimates; dots indicate individual observations, boxes represent the interquartile range, horizontal lines indicate medians, and white diamonds indicate means. Brackets show pairwise comparisons between levels of factors involved in the interactions. **(A)** Distribution of reaction times for high- and low-dominance items, separated by response type and circadian typology. **(B)** Distribution of reaction times for high- and low-dominance items presented in the left visual field (LVF) and right visual field (RVF) during morning (AM) and afternoon (PM) sessions; **(C)** Distribution of accuracy scores for high- and low-dominance items presented in the left visual field (LVF) and right visual field (RVF), separately for morning-, intermediate-, and evening-types. In each figure, **p < 0.01; ***p ≤ 0.001; ****p < 0.0001; ns, not significant.

As regards RTs, no main effects reached significance, except for Stimulus Position (*p* = 0.0001, η^2^*_*p*_* = 0.15) and Response Type (*p* = 0.0001, η^2^*_*p*_* = 0.22). Regarding the former, participants were faster at categorizing words presented in the right visual field (*M* = 1,438 ms; SD = 418 ms) compared to the left visual field (*M* = 1,477 ms; SD = 443 ms). For the latter, yes-responses (*M* = 1,417 ms; SD = 424 ms) were significantly faster than no-responses (*M* = 1,497 ms; SD = 436 ms). Among the two-way interactions (Supplementary Table 3), significant effects were found for Word Dominance × Stimulus Position (*p* = 0.025, η^2^*_*p*_* = 0.045), Word Dominance × Response Type (*p* = 0.013, η^2^*_*p*_* = 0.06), and Stimulus Position × Response Type (*p* = 0.023, η^2^*_*p*_* = 0.05). For the first interaction, Scheffè *post-hoc* tests indicated an advantage for high-dominance items in the right visual field (*M* = 1,416 ms; SD = 410 ms) relative to the left (*M* = 1,485 ms; SD = 452 ms; *p* = 0.0001), whereas no significant difference between visual fields emerged for low-dominance items (left: 1,460 ± 425 ms; right: 1,469 ± 434 ms). Regarding the second interaction, *post-hoc* tests revealed that for yes-responses, high-dominance items (*M* = 1,397 ms; SD = 411 ms) were detected faster than low-dominance items (*M* = 1,438 ms; SD = 438 ms; *p* = 0.001), while no difference was found for no-responses. The third interaction showed that for yes-responses, stimuli in the right visual field (*M* = 1,387 ms; SD = 409 ms) were judged faster than those in the left (*M* = 1,447 ms; SD = 440 ms; *p* = 0.001); no spatial differences were found for no-responses (left: 1,506 ± 446 ms; right: 1,489 ± 426 ms). Interestingly, we observed both a significant Circadian Preference × Stimulus Position interaction (*p* = 0.008, η^2^*_*p*_* = 0.09) and a Circadian Preference × Word Dominance × Response Type interaction (*p* = 0.028, η^2^*_*p*_* = 0.06). The two-way interaction showed that morning-types exhibited a slight RT advantage for stimuli in the right visual field (*M* = 1,424 ms; SD = 412 ms) compared to the left (*M* = 1,430 ms; SD = 401 ms). This advantage was more pronounced in intermediate-types (right: 1,484 ± 461 ms vs. left: 1,517 ± 480 ms; *p* = 0.001) and evening-types (right: 1,405 ± 379 ms vs. left: 1,483 ± 447 ms; *p* = 0.0001). As illustrated in Figure 3A, the three-way interaction revealed that for yes-responses, both extreme chronotypes categorized high-dominance members faster than low-dominance ones (*p* = 0.001, for all comparisons), while intermediate-types showed the opposite pattern (*p* = 0.003). For no-responses, intermediate- and evening-types were faster with low-dominance items (*p* = 0.003 for all comparisons), whereas morning-types showed no difference. Finally, a significant three-way interaction between Time-of-Day, Word Dominance, and Stimulus Position (*p* = 0.045, η^2^*_*p*_* = 0.036) was found (Figure 3B). For high-dominance members, RTs were faster in the right than in the left visual field, with this difference being larger in the afternoon (*p* = 0.0001) than in the morning (*p* = 0.007). For low-dominance members, the RT difference between visual fields was small and decreased from morning to afternoon, although no specific comparisons reached significance. No other interactions were significant.

Regarding accuracy, a specific additional pattern emerged. Significant main effects of Dominance (*p* = 0.0001, η^2^*_*p*_* = 0.23) and Response Type (*p* = 0.0001, η^2^*_*p*_* = 0.34) were found. Specifically, participants were more accurate for low-dominance members (*M* = 9.14; SD = 0.89) than for high-dominance members (*M* = 8.85; SD = 0.90). Additionally, accuracy was higher for no-responses (*M* = 9.36; SD = 0.80) than for yes-responses (*M* = 8.64; SD = 0.99). The Circadian Preference factor strongly approached statistical significance (*p* = 0.052, η^2^*_*p*_* = 0.052), suggesting, at descriptive level, that evening-types (*M* = 9.09; SD = 0.84) and morning-types (*M* = 9.04; SD = 0.87) judged more correct trials compared to intermediate-types (*M* = 8.88; SD = 0.97). The ANCOVA also revealed two significant two-way interactions. The first was Word Dominance × Stimulus Position (*p* = 0.0001, η^2^*_*p*_* = 0.43). *Post-hoc* decomposition indicated that for the LVF, participants were more accurate (*p* = 0.0001) for low-dominance members (*M* = 9.41; SD = 0.77) than for high-dominance members (*M* = 8.60; SD = 0.87). For the RVF, the opposite pattern emerged (*p* = 0.001), with higher accuracy for high-dominance members (*M* = 9.11; SD = 0.93) than for low-dominance items (*M* = 8.89; SD = 0.99). Furthermore, high-dominance members were detected more accurately in the RVF than in the LVF (*p* = 0.0001), whereas the reverse pattern was significant for low-dominance members (*p* = 0.0001). The second significant two-way interaction was Word Dominance × Response Type (*p* = 0.0001, η^2^*_*p*_* = 0.14). The *post-hoc* test showed that when a yes-response was required, participants were slightly more accurate for low-dominance members (*M* = 8.92; SD = 0.99) than for high-dominance words (*M* = 8.36; SD = 0.98; *p* = 0.0001). For no-responses, no significant differences were found between high- (*M* = 9.34; SD = 0.82) and low-dominance (*M* = 9.37; SD = 0.78) membership (*p* = 0.90). Interestingly, the Word Dominance factor tended to interact significantly with the Time-of-Day factor (*p* = 0.05, η^2^*_*p*_* = 0.04). In this case, no time-of-day differences were observed for low-dominance items (morning session: 9.15 ± 0.88; afternoon session: 9.14 ± 0.89). Conversely, high-dominance members obtained a slightly better accuracy (*M* = 8.91; SD = 0.83) than in the afternoon session (*M* = 8.79; SD = 0.97). Finally, two significant three-way interactions were observed. First, the Circadian Preference × Word Dominance × Stimulus Position interaction (*p* = 0.032, η^2^*_*p*_* = 0.06; Figure 3C) revealed, at descriptive level, that for high-dominance words presented in the LVF, evening-types (*M* = 8.79; SD = 0.78) had more number of correct trials than morning-types (*M* = 8.54; SD = 0.86) and intermediate-types (*M* = 8.46; SD = 0.96). When high-dominance members were presented in the RVF, morning-types (*M* = 9.18; SD = 0.84) had higher accuracy than the other two chronotypes (intermediate-types: 9.08 ± 0.99; evening-types: 9.06 ± 0.97). For low-dominance items in the LVF, morning- (9.48 ± 0.78) and evening-types (9.46 ± 0.64) were more accurate than intermediate-types (9.27 ± 0.90). For low-dominance items in the RVF, evening-types (*M* = 9.04; SD = 0.98) had larger number of correct trials, followed by morning-types (*M* = 8.94; SD = 1.01) and intermediate-types (*M* = 8.69; SD = 1.00). However, no significant comparisons were yielded by the *post-hoc* test. Second, the Word Dominance × Stimulus Position × Response Type interaction (*p* = 0.0001, η^2^*_*p*_* = 0.56) showed that high-dominance members requiring a yes-response were better detected when presented in the RVF (*M* = 8.94; SD = 0.97) than in the LVF (*M* = 7.78; SD = 1.00; *p* = 0.0001). The reverse pattern was observed for high-dominance members requiring a no-response, which were better detected in the LVF (*M* = 9.41; SD = 0.74) than in the RVF (*M* = 9.27; SD = 0.90), although this comparison was not significant (*p* = 0.53). Furthermore, for low-dominance members requiring a yes-response, higher accuracy was found for stimuli presented in the LVF (*M* = 9.46; SD = 0.73) than in the RVF (*M* = 8.38; SD = 1.26; *p* = 0.0001). Finally, low-dominance members requiring a no-response were better recognized when presented on the right side of the screen (*M* = 9.40; SD = 0.74) compared to the left side (*M* = 9.34; SD = 0.81), though this final comparison did not reach statistical significance (*p* = 0.29). No other main effects or significant interactions were observed.

### 3.4 Landmark task

The significant effects of the 3 × 2 × 2 × 2 mixed-design ANCOVA on mean RTs and accuracy for the vertical landmark task are reported below; the full statistical results are provided in Supplementary Table 5. Supplementary Table 4 reports mean RTs and numbers of correct trials (and relative SD) of every circadian preference tested in both sessions judging hard and easy trials presented in the left- and right-visual fields.

**FIGURE 4:**
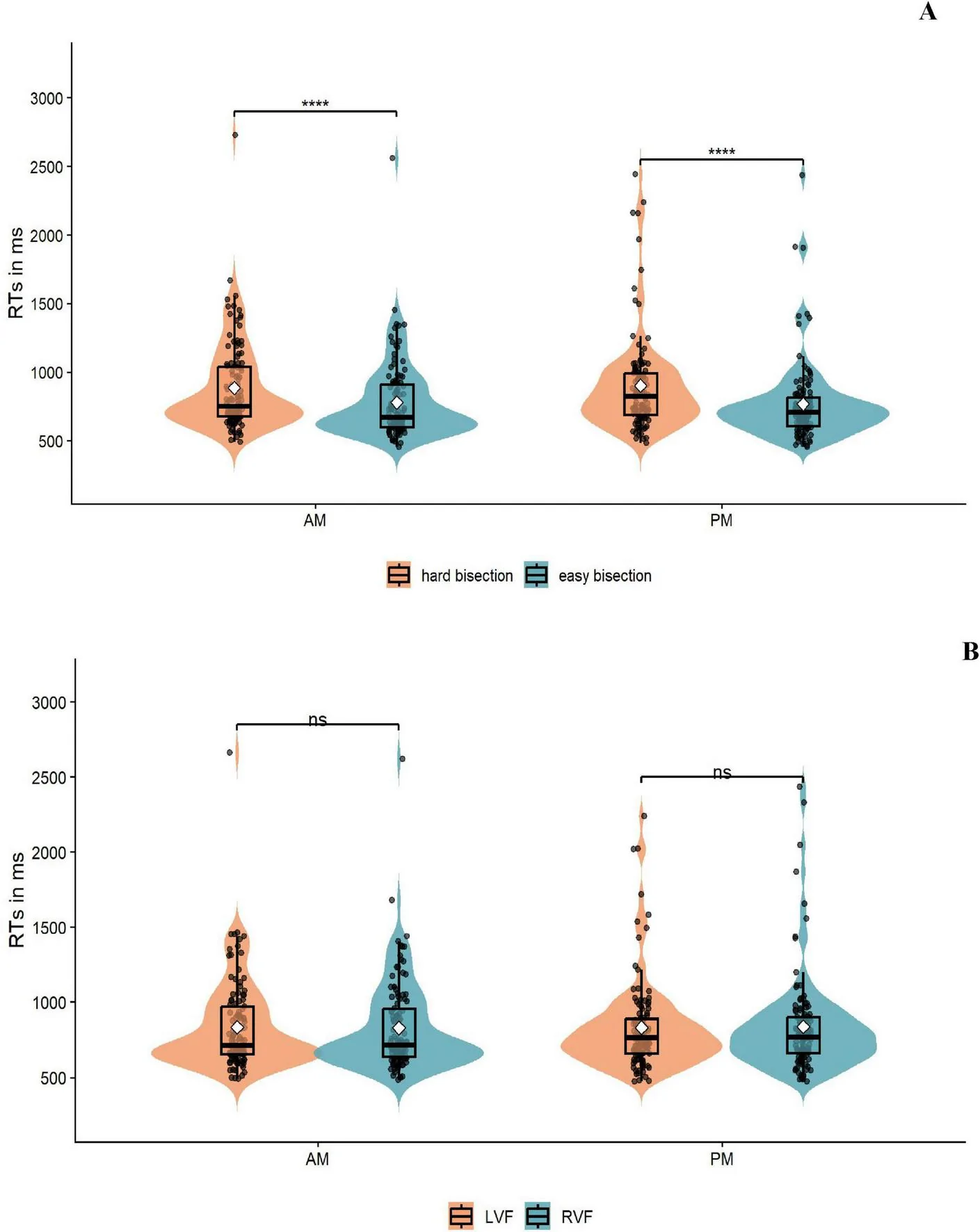
Violin plots represent kernel density estimates; dots indicate individual observations, boxes represent the interquartile range, horizontal lines indicate medians, and white diamonds indicate means. **(A)** Distribution of reaction times for hard and easy bisection trials during morning (AM) and afternoon (PM) sessions. **(B)** Distribution of reaction times for stimuli presented in the left visual field (LVF) and right visual field (RVF) during morning (AM) and afternoon (PM) sessions. In each figure, ****p < 0.0001; ns, not significant.

Regarding RTs, no main effects were observed except for a robust Task Difficulty effect (*p* = 0.00001, η^2^*_*p*_* = 0.54), indicating that easy bisection trials (*M* = 782 ms; SD = 317 ms) were significantly faster than hard trials (*M* = 901 ms; SD = 375 ms). Notably, we found significant interactions for Time-of-Day × Task Difficulty (*p* = 0.016, η^2^*_*p*_* = 0.05) and Time-of-Day × Stimulus Position (*p* = 0.045, η^2^*_*p*_* = 0.04), as shown in Figure 4. The former interaction (Figure 4A) indicated that the RT advantage for easy over hard trials was slightly greater in the afternoon (easy: *M* = 776 ms, SD = 319 ms; hard: *M* = 909 ms, SD = 393 ms; *p* = 0.0001) than in the morning (easy: *M* = 788 ms, SD = 315 ms; hard: *M* = 892 ms, SD = 356 ms; *p* = 0.0001). The latter interaction (Figure 4B) revealed that, during the morning session, participants had a quite similar performance for lines in the right visual field (*M* = 837 ms; SD = 338 ms) and the left visual field (*M* = 843 ms; SD = 333 ms). Conversely, in the afternoon, they were faster for lines in the left visual field (*M* = 838 ms; SD = 341 ms) than the right (*M* = 847 ms; SD = 372 ms), although no comparisons were statistically significant. No other interactions were found for this task.

When the accuracy was considered, a Task Difficulty effect was confirmed (*p* = 0.014, η^2^*_*p*_* = 0.054), suggesting that easy bisection trials (*M* = 9.81; SD = 0.42) were better judged than hard trials (*M* = 9.70; SD = 0.50). No other main factors and interactions were significant.

## 4 Discussion

The present study investigated the effects of time-of-day and circadian typology, as well as the synchrony effect, on semantic categorization and landmark tasks. Specifically, we examined potential variations in cognitive performance resulting from the influence of circadian and homeostatic processes on hemispheric activation throughout the day. The results partially confirmed the initial hypotheses, as detailed below. For the semantic task, we expected to replicate previous findings (Fabbri et al., 2013), with faster detection of high-dominance category members in the morning and a larger difference between high- and low-dominance items in morning-types, particularly for right visual field stimuli (left hemisphere processing). For the landmark task, we expected faster responses in the afternoon, especially for stimuli presented in the left visual field (right hemisphere processing), and superior performance in evening-types. In both tasks, the synchrony effect was expected to manifest as improved performance when testing time aligned with individual circadian preference.

In the semantic categorization task, no main effects for Time-of-Day or Circadian Preference were observed. The null effect of the Time-of-Day factor may be related to the timing of the sessions (08:00 and 13:00). Indeed, Fabbri et al. (2007) found similar retrieval efficiency in the same task for participants tested at 09:00–10:00 and 13:00–14:00, whereas efficiency changed significantly only at 17:00–18:00. At the same time, Corbera and Grau (1993) found, in a similar task, that extreme chronotypes had quite similar RTs in the morning and afternoon sessions. Beyond timing, other methodological differences might explain the results, such as the use of a within-subjects design, the recruitment of all chronotypes (rather than only extreme ones), and the lateralized visual presentation of category-item pairs, which was not employed by Fabbri et al. (2013). Nevertheless, we confirmed the lack of a synchrony effect, in agreement with May and Hasher (1998; see also Adan et al., 2012; Chauhan et al., 2025). This suggests that well-known information may be retrieved at both peak and off-peak times with minimal influence from circadian alertness (Fabbri et al., 2013). In line with this suggestion, although the Circadian Preference factor was marginally significant, descriptive values showed a tendency for extreme chronotypes to report slightly higher accuracy than intermediate-types. However, this pattern should be interpreted cautiously and requires confirmation in future studies. Interestingly, we found an interaction between circadian typology and spatial presentation (for RTs), alongside a triple interaction between circadian typology, word dominance, and response type for both speed and accuracy. The former interaction provides indirect evidence of left-hemisphere superiority in this task. Considering the main effect of Spatial Position, which indicated a RT advantage for the right visual field (left-hemisphere processing) over the left visual field (right hemisphere), all chronotypes were faster judging stimuli in the right visual field. This reflects the efficiency of left-hemisphere processing in word categorization. This preference was more pronounced in intermediate and evening types, likely because intermediate types have been possibly defined as more integrated (or balanced) thinkers (Fabbri et al., 2007), while evening types could be defined as more right-thinkers (Fabbri et al., 2007). Thus, these chronotypes appeared to benefit more from the left-hemispheric nature of the task compared to morning types, who could be typically described as left-thinkers (Fabbri et al., 2007). At the same time, this finding could confirm the associations between eveningness and verbal IQ (Killgore and Killgore, 2007) experimentally, given that our sample was completely composed of females. The RT pattern could find further support when accuracy was considered. Indeed, the Circadian Preference × Word Dominance × Spatial Position interaction indicated that high-dominance items presented in the RVF were associated with different accuracy patterns across chronotypes; however, *post-hoc* comparisons did not yield significant differences among groups (Palmero et al., 2024). Moreover, the triple interaction (Circadian Preference × Word Dominance × Response Type) could reinforce the above considerations, given that morning types (who might be associated with left-thinking style) showed faster yes-responses for high-dominance items than for low-dominance ones, with no RT differences for no-responses, suggesting superior memory efficiency (Fabbri and Martoni, 2025). The pattern observed in Figure 3A suggested a different response profile for evening-types, although these descriptive differences should be interpreted cautiously unless supported by direct statistical comparisons. This divergence aligns with models of hemispheric asymmetry in semantic processing, which propose fine or focused representations in the left hemisphere and coarse or diffuse representations in the right hemisphere (Chiarello, 1988; Deacon et al., 2004). In line with this different semantic processing of both hemispheres, we found that for yes-responses, high-dominance members were accurately detected when they were presented in the right space (left hemisphere), while low-dominance members were accurately detected when they were presented in the left space (right hemisphere). In addition, the left-hemispheric nature of the task and the possible thinking-style preferences of extreme chronotypes, according to the findings reported by Fabbri et al., 2007 (see also Corbera and Grau, 1993; Díaz-Morales and Escribano, 2013), could explain our results, with more focused semantic activation in morning types and more diffuse semantic networks in evening types, who, for example, reported higher accuracy for low-dominance items presented in the LVF. Intermediate-types (who might be associated with integrated style; Fabbri et al., 2007) showed a numerically different pattern, with relatively faster responses for low-dominance items across both response types; however, this pattern should be interpreted cautiously. Finally, we found an interaction between Time-of-Day and Word Dominance (for accuracy), which tended toward significance, and a significant interaction between Time-of-Day, Word Dominance, and Spatial Position (for RTs). The double interaction indicated that there was a slightly advantage in the morning session for high-dominance items respect to afternoon session, while no session difference was found for low-dominance items. The triple interaction reflected a greater advantage for right-visual-field pairs when judging high-dominance items from morning to afternoon, while differences in the left visual field for low-dominance items decreased during the same period. This suggests that left-hemisphere processing was favored in the morning for all pairs, whereas by the afternoon, it remained active primarily for high-dominance items (central semantic networks), suggesting a progressive deactivation of the left hemisphere in the early afternoon. Overall, these findings indicate a general involvement of the left hemisphere, and specifically left-hemisphere processing, in the morning and among morning types, partially confirming expectations (Corbera and Grau, 1993; Fabbri et al., 2007, 2013). These results may also reflect the sensitivity of the left hemisphere to the decline in cognitive performance during the early afternoon (Carrier and Monk, 2000; Craig, 1986; Czeisler and Gooley, 2007; Natale, 2002; Natale et al., 2007, 2022), supporting the two-process model and the association between left-hemispheric activity and homeostatic regulation (Borbély, 1982; Borbély et al., 2016; Rogers et al., 2003).

In the vertical landmark task (Seydell-Greenwald et al., 2017, 2019), we found no main effects for Time-of-Day or Circadian Preference, nor a synchrony effect. In addition, we marginally found what was expected. The lack of main effects aligns with previous studies showing no clear diurnal variation in visuospatial tasks and no superiority of evening types in this area (Adan et al., 2012; Chauhan et al., 2025; Dorrian et al., 2017; Natale et al., 2003; Song and Stough, 2000). Methodological choices may have also played a role; for instance, the within-subjects design might have masked main and synchrony effects, as the tasks required alternating activation of both hemispheres. Regarding the lack of significant effects for Circadian Preference, we propose that the use of a well-validated tool to categorize all chronotypes, rather than a single item (Dorrian et al., 2017), MEQ score distribution (Nishida et al., 2022), or age (Rowe et al., 2009), may account for these differences. The procedure used has previously demonstrated sensitivity in detecting differences in subjective alertness associated with circadian preference (Natale and Cicogna, 1996). However, we did find double interactions between Time-of-Day and Task Difficulty (or Spatial Position). The first interaction indicated a greater RT difference between hard and easy bisection trials in the afternoon than in the morning, suggesting a possible increased activation of the right hemisphere throughout the day. Consistent with previous vertical landmark research (Seydell-Greenwald et al., 2017, 2019), we largely replicated the Task Difficulty effect for both speed and accuracy. It has been reported that such visuospatial tasks elicit greater right-hemisphere activation due to increased demands on spatial processing, though activations remain right-lateralized across all difficulty levels (Seydell-Greenwald et al., 2017, 2019; Mesulam, 1999). Although we did not observe a definitive superiority of the left visual field over the right, the task appeared predominantly right lateralized. The more pronounced RT differences between hard and easy trials in the afternoon session may indirectly demonstrate increased right-hemisphere activation during the day (Fimm et al., 2016; Monk and Leng, 1986; van der Heijden et al., 2010). Thus, the Time-of-Day effect seemed to modulate sensitivity to perceptual demands, shaping how performance scaled with difficulty. This suggests that right-hemisphere-weighted processing fluctuates throughout the day in a graded, context-dependent manner rather than through a categorical afternoon benefit. Our findings may be consistent with those reported by Fimm et al. (2016) and Mesulam (1999), who observed a significant reduction in reaction times for both overt and covert attentional orienting across the day. Specifically, reaction times were progressively shorter during the afternoon sessions (14:00, 17:00, and 20:00) than during the morning session (08:00). However, only covert attention was significantly associated with daily fluctuations in body temperature, a marker of Process C (Borbély, 1982; Borbély et al., 2016). Although this interpretation remains speculative, these findings can be integrated with the present results by suggesting a potential relationship between Process C and right-hemisphere functioning, given the established role of the right hemisphere in visuospatial attention. Such a relationship may be particularly relevant during the afternoon and evening, when circadian arousal reaches higher levels. The Time-of-Day × Spatial Position interaction further supports this assumption. As shown in Figure 4B, participants were slightly faster judging lines in the left visual field (right hemisphere) compared to the right visual field (left hemisphere) in the afternoon, while performance was comparable in the morning. These findings align with studies demonstrating that the vertical landmark task triggers clear right-hemisphere activation, specifically in the right parietal cortex, as it primarily requires comparing the magnitude or spatial extent of two segments without involving spatial attention, motor responses, or general executive demands (Seydell-Greenwald et al., 2017, 2019). Furthermore, Fink et al. (2001) observed right-lateralized activation in the posterior parietal cortex regardless of whether lines were bisected horizontally or vertically, reinforcing the idea of a shared neural mechanism for visuospatial judgments and a general magnitude estimation system (e.g., Walsh, 2003). Altogether, these results indicate an involvement of the right hemisphere with greater activation during the day (see also Corbera and Grau, 1993), partially aligning with expected findings (Dorrian et al., 2017; Manly et al., 2005; Monk and Leng, 1986; van der Heijden et al., 2010). This may reflect the sensitivity of the right hemisphere to Process C, with peak activation of the right parietal hemisphere in the early afternoon supporting the two-process model and circadian arousal fluctuations (Borbély, 1982; Borbély et al., 2016; Colquhoun, 1971; Rogers et al., 2003).

The study is not without limitations. A limitation of the present study concerns the categorization of chronotype based on rMEQ-derived cut-off scores. Although this classification approach is widely used in chronobiological research and allows the comparison of distinct circadian preference profiles based on established rMEQ scoring criteria rather than on data-driven cut-offs, it may reduce the information contained in the original continuous measure and may not fully capture the gradual nature of individual differences in morningness–eveningness, warranting caution in interpretations. Future studies may complement categorical approaches with analyses considering chronotype as a continuous dimension to further characterize the relationship between circadian preference and cognitive performance. Since we only selected two testing points, future research should include additional sessions across the day, widening the interval (e.g., including a late-evening assessment) to better capture circadian and homeostatic dynamics. Indeed, the lack of Time-of-Day or Circadian Preference effects on body temperature was likely due to this narrow temporal window, although our testing sessions were sufficient to capture changes in subjective vigor in different circadian preference. Implementing an evening session near bedtime and wake-up times could better capture variations in hemispheric activation (Dorrian et al., 2017; Fabbri et al., 2013; Natale et al., 2022). Additionally, larger samples with balanced sex distribution are required to ensure generalizability. To control for documented sex differences in cognitive performance, lateralization, and chronotype distribution, only females were enrolled; future studies should include male participants in balanced proportions. As the sample consisted exclusively of female participants, a potential source of variability may relate to the menstrual cycle phase and the use of hormonal contraceptives. Indeed, fluctuations in ovarian hormones have been associated with variations in visuospatial performance (e.g., Maki et al., 2002) and functional lateralization (e.g., Wong-Goodrich et al., 2020). Thus, future studies should record and control for the menstrual cycle phase, even though research on menstrual cycle-dependent cognitive changes has yielded inconsistent results, suggesting that such effects on cognitive functions are relatively small (e.g., Sundström Poromaa and Gingnell, 2014). Furthermore, circadian and homeostatic influences were primarily assessed through self-reports and indirect proxies. Incorporating independent circadian phase measures and objective physiological markers, such as continuous body temperature, melatonin, cortisol levels, or motor activity recorded through actigraphy, is essential for a more robust study. Methodologically, we acknowledge that the absence of eye-tracking or formal fixation monitoring represents a limitation of this study. In our study, stimuli remained on screen until the participant’s response, and eye movements could not be prevented. While the use of a chin and forehead rest effectively stabilized head position, we could not completely rule out saccadic eye movements and potential gaze shift. Thus, this methodological aspect could question fixation to distinguish subtle hemispheric and chronobiological influences from generic alertness or motor components, increasing the reliability of higher-order interaction effects. In addition, although reaction times were analyzed at the condition level, a trial-level linear mixed effects approach could provide a more sensitive framework by accounting for within-participant variability and trial-by-trial fluctuations. The absence of such analysis represents a limitation of the present study, as trial-level modeling may help distinguish subtle experimental effects from unsystematic variability and provide a more robust assessment of higher-order interactions. Future studies should apply trial-level mixed-effects models to further validate the present findings. However, the specific pattern of results could suggest that hemispheric processing remained a factor, though the findings should be interpreted with caution regarding strict lateralization. Moreover, a limitation of the present study is that individual food and caffeine intake was not strictly quantified through objective logs. Although participants were requested to avoid consumption 1 h prior to testing and the counterbalanced order of session mitigated systematic biases, future studies should strictly control or standardize nutritional intake to isolate pure circadian fluctuations. Finally, we acknowledge that certain effect sizes were small, highlighting the need for future studies to validate these results. In addition, especially for semantic categorization task, a possible speed-accuracy trade-off could be advanced, although we decided to include in the data analysis participants with an accuracy higher than 80 only. Despite of these limitations, the present data could be applied in specific settings, such as school, given that time-of-day and morningness-eveningness preferences (with a possible association with thinking styles) influence academic achievement (e.g., Díaz-Morales and Escribano, 2013; Zerbini and Merrow, 2017). For example, van der Heijden et al. (2010) found, in preadolescents, time-of-day effects on cognitively more demanding tasks (i.e., visuospatial processing and working memory), requiring the processing of input and central cognitive performance. At the same time, Díaz-Morales and Escribano (2013) reported in preadolescents and adolescents that morning-types with left-thinking reported the highest subjective school achievement, followed by evening-types with left-thinking, and morning-types with right-thinking, whereas evening-types with right-thinking reported the lowest subjective level of achievement.

## 5 Conclusion

In conclusion, this study investigated the effects of time of day, circadian typology, and their interaction on semantic categorization and visuospatial processing. Through these two tasks, we found partial support for the association between the left hemisphere and homeostatic processes, and between the right hemisphere and circadian processes, with primary left-hemisphere activation in the morning and right-hemisphere activation in the afternoon. Chronotypes appeared to modulate performance only in the semantic categorization task, probably supporting the previous associations between thinking styles and chronotypes, especially between left-thinking style and morning types. The absence of circadian typology modulation in the landmark task, contrary to some literature, may stem from the specific methodology used to categorize individuals.

## Data Availability

The raw data supporting the conclusions of this article will be made available by the authors, without undue reservation.

## References

[B1] Adan A. Almirall H. (1991). Horne and Östberg Morningness-eveningness questionnaire: A reduced scale. *Pers. Individ. Dif.* 12 241–253. 10.1016/0191-8869(91)90110-w

[B2] Adan A. Archer S. N. Hidalgo M. P. Di Milia L. Natale V. Randler C. (2012). Circadian typology: A comprehensive review. *Chronobiol. Int.* 29 1153–1175. 10.3109/07420528.2012.719971 23004349

[B3] Adan A. Prat G. Fabbri M. Sànchez-Turet M. (2008). Early effects of caffeinated and decaffeinated coffee on subjective state and gender differences. *Prog. Neuro-Psychopharmacol. Biol. Psychiatry* 32 1698–1703. 10.1016/j.pnpbp.2008.07.005 18675877

[B4] Anderson M. J. Petros T. V. Beckwith B. E. Mitchell W. W. Fritz S. (1991). Individual differences in the effect of time of day on long-term memory access. *Am. J. Psychol.* 104 241–255. 10.2307/1423157 27413571 PMC4931098

[B5] Beeman M. J. (1998). “Coarse semantic coding and discourse comprehension,” in *Right Hemisphere Language Comprehension: Perspectives from Cognitive Neuroscience*, eds Beeman M. J. Chiarello C. (Mahwah, NJ: Lawrence Erlbaum Associates), 255–284.

[B6] Bettencourt C. Pires L. Vilar M. Almeida F. Samarra S. Duarte R. et al. (2025). Circadian dynamics of explicit memory performance in youth: Exploring chronotype and synchrony effects. *Memory* 33 896–908. 10.1080/09658211.2025.2536688 40684440

[B7] Blatter K. Cajochen C. (2007). Circadian rhythms in cognitive performance: Methodological constraints, protocols, theoretical underpinnings. *Physiol. Behav.* 90 196–208. 10.1016/j.physbeh.2006.09.009 17055007

[B8] Borbé,ly A. A. (1982). A two process model of sleep regulation. *Hum. Neurobiol.* 1 195–204. 7185792

[B9] Borbély A. A. Daan S. Wirz-Justice A. Deboer T. (2016). The two-process model of sleep regulation: A reappraisal. *J. Sleep Res.* 25 131–143. 10.1111/jsr.12371 26762182

[B10] Carrier J. Monk T. H. (2000). Circadian rhythms of performance: New trends. *Chronobiol. Int.* 17 719–732. 10.1081/cbi-100102108 11128289

[B11] Ceglarek A. Hubalewska-Mazgaj M. Lewandowska K. Sikora-Wachowicz B. Marek T. Fafrowicz M. (2021). Time-of-day effects on objective and subjective short-term memory task performance. *Chronobiol. Int.* 38 1330–1343. 10.1080/07420528.2021.1929279 34121547

[B12] Chauhan S. Vanova M. Tailor U. Asad M. Faβbender K. Norbury R. et al. (2025). Chronotype and synchrony effects in human cognitive performance: A systematic review. *Chronobiol. Int.* 42 463–499. 10.1080/07420528.2025.2490495 40293205

[B13] Chiarello C. (1988). *Right Hemisphere Contributions to Lexical Semantics.* Berlin, NY: Springer-Verlag.

[B14] Çiçek M. Deouell L. Y. Knight R. T. (2009). Brain activity during landmark and line bisection tasks. *Front. Hum. Neurosci.* 3:7. 10.3389/neuro.09.007.2009 19521543 PMC2694675

[B15] Colquhoun W. P. (1971). “Circadian variation in mental efficiency,” in *Biological Rhythms and Human Performance*, ed. Colquhoun W. P. (London: Academic Press), 39–55.

[B16] Corbera X. Grau C. (1993). Diurnal type and hemispheric asymmetry. *Cortex* 29 519–528. 10.1016/S0010-9452(13)80257-2 8258289

[B17] Corbera X. Grau C. Vendrell P. (1993). Diurnal oscillations in hemispheric performance. *J. Clin. Exp. Neuropsychol.* 15 300–310. 8491852 10.1080/01688639308402564

[B18] Correa A. Ruiz-Herrera N. Ruz M. Tonetti L. Martoni M. Fabbri M. et al. (2017). Economic decision-making in morning/evening-type people as a function of time of day. *Chronobiol. Int.* 34 139–147. 10.1080/07420528.2016.1246455 27791397

[B19] Craig A. (1986). Acute effects of meals on perceptual and cognitive efficiency. *Nutr. Rev.* 44, 163–171. 10.1111/j.1753-4887.1986.tb07693.x 2980847

[B20] Czeisler C. A. Gooley J. J. (2007). Sleep and circadian rhythms in humans. *Cold Spring Harb. Symp. Quant. Biol.* 72 579–597. 10.1101/sqb.2007.72.064 18419318

[B21] Deacon D. Grose-Fifer J. Yang C. M. Stanick V. Hewitt S. Dynowska A. (2004). Evidence for a new conceptualization of semantic representation in a left and right cerebral hemispheres. *Cortex* 40 467–478. 10.1016/s0010-9452(08)70140-0 15259327

[B22] Díaz-Morales J. F. Escribano C. (2013). Circadian preference and thinking styles: Implications for school achievement. *Chronobiol. Int.* 30 1231–1239. 10.3109/07420528.2013.813854 24024592

[B23] Dorrian J. McLean B. Banks S. Loetscher T. (2017). Morningness/eveningness and the synchrony effect for spatial attention. *Accid. Anal. Prev.* 99 401–405. 10.1016/j.aap.2015.11.012 26775078

[B24] Fabbri M. Martoni M. (2025). How chronotype, sleep-wake cycle, subjective time experience influence retrospective and prospective memory functioning. *Front. Cognit.* 4:1683207. 10.3389/fcogn.2025.1683207 42339246 PMC13281119

[B25] Fabbri M. Antonietti A. Giorgetti M. Tonetti L. Natale V. (2007). Circadian typology and style of thinking differences. *Learn. Individ. Dif.* 17 175–180. 10.1016/j.lindif.2007.05.002

[B26] Fabbri M. Frisoni M. Martoni M. Tonetti L. Natale V. (2017). Synchrony effect on joint attention. *Exp. Brain Res.* 235 2449–2462. 10.1007/s00221-017-4984-6 28509111

[B27] Fabbri M. Mencarelli C. Adan A. Natale V. (2013). Time-of-day and circadian typology on memory retrieval. *Biol. Rhythm Res.* 44 125–142. 10.1080/09291016.2012.656244

[B28] Fimm B. Brand T. Spijkers W. (2016). Time-of-day variation of visuo-spatial attention. *Br. J. Psychol.* 107 299–321. 10.1111/bjop.12143 26248950

[B29] Fink G. R. Marshall J. C. Weiss P. H. Zilles K. (2001). The neural basis of vertical and horizontal line bisection judgments. An fMRI study of normal volunteers. *NeuroImage* 14 S59–S67. 10.1006/nimg.2001.0819 11373134

[B30] Gur R. C. Gur R. E. (2017). Complementarity of sex differences in brain and behavior: From laterality to multi-modal neuroimaging. *J. Neurosci. Res.* 95 189–199. 10.1002/jnr.23830 27870413 PMC5129843

[B31] Hausmann M. Ergun G. Yazgan Y. Güntürkün O. (2002). Sex differences in line bisection as a function of hand. *Neuropsychologia* 40 235–240. 10.1016/s0028-3932(01)00112-9 11684155

[B32] Heber I. A. Stebertz S. Wolter M. Kuhlen T. Finn B. (2010). Horizontal and vertical pseudoneglect in peri- and extrapersonal space. *Brain Cogn.* 73 160–166. 10.1016/j.bandc.2010.04.006 20537456

[B33] Hidalgo M. P. L. Zanette C. B. Pedrotti M. Souza C. M. Nunes P. V. Chaves M. L. F. (2004). Performance of chronotypes on memory tests during the morning and the evening shifts. *Psychol. Rep.* 95 75–85. 15460360 10.2466/pr0.95.1.75-85

[B34] Horne J. A. Östberg O. (1976). A self-assessment questionnaire to determine morningness-eveningness in human circadian rhythms. *Int. J. Chronobiol.* 4 97–110. 1027738

[B35] Jewell G. McCourt M. E. (2000). Pseudoneglect: A review and meta-analysis of performance factors in line bisection tasks. *Neuropsychologia* 38 93–110. 10.1016/s0028-3932(99)00045-7 10617294

[B36] Killgore W. D. Killgore D. B. (2007). Morningness-eveningness correlates with verbal ability in women not men. *Percept. Mot. Skills* 104 335–338. 10.2466/pms.104.1.335-338 17450994

[B37] Mahoney C. R. Taylor H. A. Kanarek R. B. (2005). “The acute effects of meals on cognitive performance,” in *Nutritional Neuroscience*, eds Lieberman H. R. Kanarek R. B. Prasad C. (Milton Park: Taylor & Francis), 73–91. 10.1201/9780203564554.ch6

[B38] Maki P. M. Rich J. B. Rosenbaum R. S. (2002). Implicit memory varies across the menstrual cycle: Estrogen effects in young women. *Neuropsychologia* 40 518–552. 10.1016/S0028-3932(01)00126-9 11749982

[B39] Manly T. Dobler V. B. Dodds C. M. George M. A. (2005). Rightward shift in spatial awareness with declining alertness. *Neuropsychologia* 43 1721–1728. 10.1016/j.neuropsychologia.2005.02.009 16154447

[B40] May C. P. Hasher L. (1998). Synchrony effects in inhibitory control thought and action. *J. Exp. Psychol. Hum. Percept. Perform.* 24 363–379. 10.1037//0096-1523.24.2.363 9554091

[B41] May C. P. Hasher L. Healey K. (2023). For whom (and when) the time bell tolls: Chronotypes and the synchrony effect. *Perspect. Psychol. Sci.* 18 1520–1536. 10.1177/17456916231178553 37369064

[B42] Mesulam M. M. (1999). Spatial attention and neglect: Parietal, frontal and cingulate contributions to the mental representation and attentional targeting of salient extrapersonal events. *Philos. Trans. R. Soc. Lond. B Biol. Sci.* 354 1325–1346. 10.1098/rstb.1999.0482 10466154 PMC1692628

[B43] Mongrain V. Carrier J. Paquet J. Bélanger-Nelson E. Dumont M. (2011). Morning and evening-type differences in slow waves during NREM sleep reveal both trait and state dependent phenotypes. *PLoS One* 6:e22679. 10.1371/journal.pone.0022679 21829643 PMC3150370

[B44] Monk T. H. (1989). A Visual Analogue Scale technique to measure global vigor and affect. *Psychiatry Res.* 27 89–99. 10.1016/0165-1781(89)90013-9 2922449

[B45] Monk T. H. Leng V. C. (1986). Interactions between inter-individual and inter-task differences in the diurnal variation of human performance. *Chronobiol. Int.* 3 171–177. 10.3109/07420528609066364 3677200

[B46] Natale V. (1999). Validazione di una scala ridotta di Mattutinità (rMEQ). *Boll. Psicol. Appl.* 229 19–26.

[B47] Natale V. (2002). Circadian motor asymmetries in humans. *Neurosci. Lett.* 320 102–104. 10.1016/s0304-3940(02)00031-9 11849774

[B48] Natale V. Alzani A. Cicogna P. C. (2003). Cognitive efficiency and circadian typology: A diurnal study. *Pers. Individ. Dif.* 35 1089–1105. 10.1016/S0191-8869(02)00320-3

[B49] Natale V. Cicogna P. C. (1996). Circadian regulation of subjective alertn-ess in morning and, evening type. *Pers. Individ. Dif.* 20 491–497. 10.1016/0191-8869(95)00213-8

[B50] Natale V. Fabbri M. Martoni M. Tonetti L. (2022). Circadian motor activity of non-dominant hand reaches acrophase later than dominant hand. *Sci. Rep.* 12:5748. 10.1038/s41598-022-09717-5 35388093 PMC8987093

[B51] Natale V. Martoni M. Esposito M. J. Fabbri M. Tonetti L. (2007). Circadian motor asymmetries before and after prolonged wakefulness in humans. *Neurosci. Lett.* 423 216–218. 10.1016/j.neulet.2007.07.026 17703883

[B52] Nishida M. Ando H. Murata Y. Shioda K. (2022). Mental rotation performance and circadian chronotype in university students: A preliminary study. *Biol. Rhythm Res.* 53 1030–1042. 10.1080/09291016.2021.1890366

[B53] Oldfield R. C. (1971). The assessment and analysis of handedness: The Edinburgh Inventory. *Neuropsychologia* 9 97–113. 10.1016/0028-3932(71)90067-4 5146491

[B54] Palmero L. B. Tortajada M. Martínez-Pérez V. Sandoval-Lentisco A. Campoy G. Fuentes L. J. (2024). Circadian modulation of the time course of automatic and controlled semantic processing. *Chronobiol. Int.* 41 378–392. 10.1080/07420528.2024.2312806 38317372

[B55] Pasterski V. Zwierzynska K. Estes Z. (2011). Sex differences in semantic categorization. *Arch. Sex Behav.* 40 1183–1187. 10.1007/s10508-011-9764-y 21516365

[B56] Petros T. Beckwith W. Anderson M. (1990). Individual differences in the effect of time of day and passage of difficulty on prose memory in adults. *Br. J. Psychol.* 81 63–72. 10.1111/j.2044-8295.1990.tb02346.x

[B57] Putilov A. A. Donskaya O. G. (2022). What can make the difference between chronotypes in sleep duration? Testing the similarity of their homeostatic processes. *Front. Neurosci.* 16:832807. 10.3389/fnins.2022.832807 35299620 PMC8920995

[B58] Rogers N. L. Dorrian J. Dingers D. F. (2003). Sleep, waking and neurobehavioural performance. *Front. Biosci.* 8:S1056–S1067. 10.2741/1174 12957855

[B59] Rowe G. Hasher L. Turcotte J. (2009). Age and synchrony effects in visuospatial working memory. *Q. J. Exp. Psychol.* 1873–1880. 10.1080/17470210902834852 19459136

[B60] Schmidt C. Collette F. Cajochen C. Peigneux P. (2007). A time to think: Circadian rhythms in, human cognition. *Cogn. Neuropsychol.* 24 755–789. 10.1080/02643290701754158 18066734

[B61] Schmidt C. Peigneux P. Leclercq Y. Sterpenich V. Vanderwalle G. Phillips C. et al. (2012). Circadian preference modulates the neural substrate of conflict processing across the day. *PLoS One* 7:e29658. 10.1371/journal.pone.0029658 22238632 PMC3251569

[B62] Schneider W. Eschman A. Zuccolotto A. (2012). *E-Prime User’s Guide.* Pittsburgh, PA: Psychology Software Tools, Inc.

[B63] Seydell-Greenwald A. Ferrara K. Chambers C. E. Newport E. L. Landau B. (2017). Bilateral parietal activation for complex visual-spatial functions: Evidence from a visual-spatial construction task. *Neuropsychologia* 106 194–206. 10.1016/j.neuropsychologia.2017.10.005 28987904 PMC6408728

[B64] Seydell-Greenwald A. Pu S. F. Ferrara K. Chambers C. E. Newport E. L. Landau B. (2019). Revisiting the Landmark Task as a tool for studying hemispheric specialization: What’s really right? *Neuropsychologia* 127 57–65. 10.1016/j.neuropsychologia.2019.01.022 30802463 PMC6440843

[B65] Song J. Stough C. (2000). The relationship between Morningness-Eveningness, time-of-day, speed of information processing, and intelligence. *Pers. Individ. Dif.* 29 1179–1190. 10.1016/S0191-8869(00)00002-7

[B66] Sundström Poromaa I. Gingnell M. (2014). Menstrual cycle influence on cognitive function and emotion processing from a reproductive perspective. *Front. Neurosci.* 8:380. 10.3389/fnins.2014.00380 25505380 PMC4241821

[B67] Taillard J. Philip P. Coste O. Sagaspe P. Bioulac B. (2003). The circadian and homeostatic modulation of sleep pressure during wakefulness differ between morning and evening chronotypes. *J. Sleep Res.* 12 275–282. 10.1046/j.0962-1105.2003.00369.x 14633238

[B68] Tilley A. Warren P. (1983). Retrieval from semantic memory at different times of day. *J. Exp. Psychol. Learn. Mem. Cogn.* 9 718–724. 10.1037//0278-7393.9.4.718 6227684

[B69] Tonetti L. Fabbri M. Natale V. (2008). Sex difference in sleep-time preference and sleep need: A cross-sectional survey among Italian pre-adolescents, adolescents, and adults. *Chronobiol. Int.* 25 745–759. 10.1046/j.1442-200X.2001.01466.x 18780201

[B70] van der Heijden K. B. de Sonneville L. M. J. Althaus M. (2010). Time-of-day effects on cognition in preadolescents: A trials study. *Chronobiol. Int.* 27 1870–1894. 10.3109/07420528.2010.516047 20969529

[B71] Van Dongen H. P. Dinges D. F. (2003). Investigating the interaction between the homeostatic and circadian processes of sleep-wake regulation for the prediction of waking neurobehavioural performance. *J. Sleep Res.* 12 181–187. 10.1046/j.1365-2869.2003.00357.x 12941057

[B72] Van Dongen H. P. Dinges D. F. (2005). “Circadian rhythms in sleepiness, alertness, and performance,” in *Principles and Practice of Sleep Medicine, fourth edition*, Chap. 35, eds Kryger M. H. Roth T. Dement W. C. (Philadelphia: Saunders Elsevier), 435–443. 10.1016/B0-72-160797-7/50042-2

[B73] Van Opstal F. Aslanov V. Schnelzer S. (2022). Mind-wandering in larks and owls: The effect of chronotype and time of day on the frequency of task-unrelated thoughts. *Collabra Psychol.* 8:1. 10.1525/Collabra.57536

[B74] Walsh V. (2003). A theory of magnitude: Common cortical metrics of time, space, and quantity. *Trends Cogn Sci* 7 483–488. 10.1016/j.tics.2003.09.002 14585444

[B75] Wiłkość-Dêbczyńska M. Liberacka-Dwojak M. (2023). Time of day and chronotype in the assessment of cognitive functions. *Postep. Psychiatr. Neurol.* 32 162–166. 10.5114/ppn.2023.132032 38034504 PMC10683050

[B76] Wong-Goodrich S. J. E. DeRosa H. J. Kee D. W. (2020). Dual-task paradigm reveals variation in left hemisphere involvement in verbal processing across the menstrual cycle in normally cycling women. *Psychol. Rep.* 123 2372–2393. 10.1177/0033294119862992 31291167

[B77] Wright K. P. Hull J. T. Czeisler C. A. (2002). Relationship between alertness, performance, and body temperature in humans. *Am. J. Physiol. Regul. Integr. Comp. Physiol.* 283 R1370–R1377. 10.1152/ajpregu.00205.2002 12388468

[B78] Zerbini G. Merrow M. (2017). Time to learn: How chronotype impacts education. *Psych J.* 6 263–276. 10.1002/pchj.178 28994246

