# Supplementary material for: Hemispheric efficiency during the day: the influence of circadian preference and time-of-day on verbal and visuospatial tasks
Source: Front Psychol. 2026 Sep 3;17:1885699. doi: 10.3389/fpsyg.2026.1885699 (PMC13582664; doi:10.3389/fpsyg.2026.1885699)
Supplement: Supplementary file 1 [file Table_1.docx]

Supplementary Material

In the Supplementary Table 1 (S1), the means and relative standard deviations of objective (body temperature in °C) and subjective (GVA vigor score) alertness are reported.

**Table S1**. The mean (M) and relative standard deviation (SD) of body temperature (in °C) and GVA vigor score in both testing times for each circadian preference.

|  | Body Temperature | | | | GVA Vigor Score | | | |
| --- | --- | --- | --- | --- | --- | --- | --- | --- |
|  | **Morning Session** | | **Afternoon Session** | | **Morning Session** | | **Afternoon Session** | |
|  | **M** | **SD** | **M** | **SD** | **M** | **SD** | **M** | **SD** |
| Evening-types  (n = 20) | 35.24 | 0.39 | 35.27 | 0.52 | 43.48 | 19.58 | 63.19 | 16.89 |
| Intermediate-types  (n = 67) | 35.13 | 0.35 | 35.28 | 0.36 | 54.54 | 16.70 | 64.70 | 16.46 |
| Morning-types  (n = 26) | 35.58 | 1.77 | 35.33 | 0.37 | 63.14 | 18.42 | 66.71 | 14.72 |
| Total sample | 35.32 | 0.84 | 35.29 | 0.42 | 53.72 | 18.23 | 64.87 | 16.02 |

The mean RTs and number of correct trials (and relative SDs) in the semantic categorization task are reported in Table S2.

**Table S2.** The mean RTs in ms and numbers of correct trials, defined as accuracy, (and relative SDs) of semantic categorization task performed in both testing sessions by each circadian preferences, for high- and low-dominance items presented in the left- and right-visual field (LVF and RVF respectively) requiring yes- and no-responses.

| RTs | Morning Session | | | | | | | | Afternoon Session | | | | | | | |
| --- | --- | --- | --- | --- | --- | --- | --- | --- | --- | --- | --- | --- | --- | --- | --- | --- |
|  | **High-dominance** | | **Low-dominance** | | **High-dominance** | | **Low-dominance** | | **High-dominance** | | **Low-dominance** | | **High-dominance** | | **Low-dominance** | |
|  | **LVF** | | **LVF** | | **RVF** | | **RVF** | | **LVF** | | **LVF** | | **RVF** | | **RVF** | |
|  | **Yes** | **No** | **Yes** | **No** | **Yes** | **No** | **Yes** | **No** | **Yes** | **No** | **Yes** | **No** | **Yes** | **No** | **Yes** | **No** |
| Evening-types | 1449  (486) | 1557  (416) | 1551  (604) | 1526  (384) | 1346  (375) | 1510  (438) | 1437  (385) | 1469  (336) | 1373  (326) | 1537  (588) | 1418  (339) | 1456  (433) | 1226  (245) | 1423  (416) | 1356  (386) | 1471  (452) |
| Intermediate-types | 1513  (543) | 1579  (536) | 1436  (470) | 1545  (554) | 1426  (499) | 1540  (507) | 1494  (548) | 1556  (536) | 1572  (478) | 1547  (449) | 1447  (393) | 1494  (420) | 1394  (373) | 1504  (436) | 1439  (392) | 1522  (400) |
| Morning-types | 1339  (310) | 1422  (367) | 1354  (323) | 1440  (396) | 1325  (374) | 1446  (394) | 1374  (380) | 1436  (353) | 1441  (481) | 1485  (440) | 1476  (524) | 1482  (368) | 1356  (440) | 1494  (426) | 1474  (507) | 1491  (421) |
|  | | | | | | | | | | | | | | | | |
| Numbers of Correct Trials | **Morning Session** | | | | | | | | **Afternoon Session** | | | | | | | |
|  | **High-dominance** | | **Low-dominance** | | **High-dominance** | | **Low-dominance** | | **High-dominance** | | **Low-dominance** | | **High-dominance** | | **Low-dominance** | |
|  | **LVF** | | **LVF** | | **RVF** | | **RVF** | | **LVF** | | **LVF** | | **RVF** | | **RVF** | |
|  | **Yes** | **No** | **Yes** | **No** | **Yes** | **No** | **Yes** | **No** | **Yes** | **No** | **Yes** | **No** | **Yes** | **No** | **Yes** | **No** |
| Evening-types | 8.05  (0.83) | 9.55  (0.69) | 9.40  (0.78) | 9.50  (0.61) | 8.90  (1.02) | 9.25  (0.97) | 8.55  (1.32) | 9.25  (0.85) | 8.10  (0.91) | 9.45  (0.69) | 9.80  (0.41) | 9.15  (0.75) | 9.05  (0.69) | 9.05  (1.19) | 8.95  (1.05) | 9.40  (0.68) |
| Intermediate-types | 7.58  (1.16) | 9.37  (0.87) | 9.19  (0.89) | 9.33  (0.77) | 9.01  (0.98) | 9.34  (0.64) | 8.12  (1.20) | 9.24  (0.85) | 7.67  (0.98) | 9.21  (0.84) | 9.30  (0.92) | 9.27  (1.02) | 8.70  (1.33) | 9.27  (1.02) | 8.04  (1.27) | 9.36  (0.71) |
| Morning-types | 7.69  (0.88) | 9.62  (0.50) | 9.65  (0.59) | 9.54  (0.76) | 9.19  (0.80) | 9.42  (0.64) | 8.46  (1.21) | 9.58  (0.76) | 7.58  (1.24) | 9.27  (0.83) | 9.46  (0.81) | 9.27  (0.96) | 8.81  (0.98) | 9.31  (0.93) | 8.15  (1.49) | 9.58  (0.58) |

In Table S3, the statistical results of the mixed ANCOVA on mean RTs and accuracy for the semantic categorization task are reported.

**Table S3.** The F value, the degrees of freedom (Df), p value and partial eta-squared (ƞ^2^_p_) are reported for main effects and all interactions. In bold the statistically significant results, while in italics results approaching statistical significance.

|  | RTs | | | | Accuracy | | | |
| --- | --- | --- | --- | --- | --- | --- | --- | --- |
|  | *F* | Df | *p* | *ƞ^2^_p_* | *F* | Df | *p* | *ƞ^2^_p_* |
| *Circadian Preference* | 0.52 | 2,109 | .60 | .009 | *3.02* | *2,109* | *.053* | *.052* |
| Time-of-Day | 0.001 | 1,109 | .98 | .0001 | 1.09 | 1,109 | .30 | .012 |
| Dominance | 1.33 | 1,109 | .25 | .012 | **31.98** | **1,109** | **.0001** | **.23** |
| Stimulus Position | **19.03** | **1,109** | **.0001** | **.15** | 0.001 | 1,109 | .97 | .0001 |
| Response Type | **30.15** | **1,109** | **.0001** | **.22** | **56.26** | **1,109** | **.0001** | **.34** |
| Circadian Preference x Time-of-Day | 0.62 | 2,109 | .54 | .011 | 1.27 | 2,109 | .29 | .02 |
| Circadian Preference x Dominance | 2.80 | 2,109 | .07 | .05 | 0.68 | 2,109 | .51 | .012 |
| Circadian Preference x Stimulus Position | **5.08** | **2,109** | **.008** | **.09** | 1.59 | 2,109 | .21 | .03 |
| Circadian Preference x Response Type | 0.56 | 2,109 | .58 | .01 | 2.72 | 2,109 | .07 | .05 |
| *Time-of-Day x Dominance* | 0.15 | 1,109 | .70 | .001 | *3.94* | *1,109* | *.05* | *.04* |
| Time-of-Day x Stimulus Position | 0.15 | 1,109 | .70 | .001 | 0.31 | 1,109 | .58 | .003 |
| Time-of-Day x Response Type | 0.11 | 1,109 | .74 | .001 | 0.84 | 1,109 | .36 | .008 |
| Dominance x Stimulus Position | **5.16** | **1,109** | **.025** | **.045** | **82.63** | **1,109** | **.0001** | **.43** |
| Dominance x Response Type | **6.44** | **1,109** | **.013** | **.06** | **17.69** | **1,109** | **.0001** | **.14** |
| Stimulus Position x Response Type | **5.34** | **1,109** | **.023** | **.05** | 2.21 | 1,109 | .14 | .02 |
| Circadian Preference x Time-of-Day x Dominance | 0.50 | 2,109 | .61 | .009 | 0.09 | 2,109 | .92 | .002 |
| Circadian Preference x Time-of-Day x Stimulus Position | 0.60 | 2,109 | .55 | .011 | 1.05 | 2,109 | .35 | .02 |
| Circadian Preference x Time-of-Day x Response Type | 1.28 | 2,109 | .28 | .02 | 1.10 | 2,109 | .34 | .02 |
| Circadian Preference x Dominance x Stimulus Position | 2.00 | 2,109 | .14 | .035 | **3.55** | **2,109** | **.032** | **.06** |
| Circadian Preference x Dominance x Response Type | **3.69** | **2,109** | **.028** | **.06** | 0.40 | 2,109 | .67 | .007 |
| Circadian Preference x Stimulus Position x Response Type | 0.13 | 2,109 | .88 | .002 | 0.78 | 2,109 | .46 | .014 |
| Time-of-Day x Dominance x Stimulus Position | **4.12** | **1,109** | **.045** | **.036** | 1.34 | 1,109 | .25 | .012 |
| Time-of-Day x Dominance x Response Type | 0.69 | 1,109 | .41 | .006 | 0.03 | 1,109 | .87 | .0001 |
| Time-of-Day x Stimulus Position x Response Type | 0.26 | 1,109 | .61 | .002 | 3.23 | 1,109 | .08 | .03 |
| Dominance x Stimulus Position x Response Type | 1.65 | 1,109 | .20 | .015 | **138.20** | **1,109** | **.0001** | **.56** |
| Circadian Preference x Time-of-Day x Dominance  x Stimulus Position | 0.76 | 2,109 | .47 | .01 | 0.94 | 2,109 | .40 | .017 |
| Circadian Preference x Time-of-Day x Dominance  x Response Type | 0.15 | 2,109 | .86 | .003 | 0.39 | 2,109 | .68 | .007 |
| Circadian Preference x Time-of-Day x Stimulus Position  x Response Type | 0.56 | 2,109 | .57 | .01 | 1.23 | 2,109 | .30 | .022 |
| Circadian Preference x Dominance x Stimulus Position  x Response Type | 0.82 | 2,109 | .45 | .015 | 0.23 | 2,109 | .80 | .004 |
| Time-of-Day x Dominance x Stimulus Position x Response Type | 0.06 | 1,109 | .81 | .001 | 0.49 | 1,109 | .49 | .004 |
| Circadian Preference x Time-of-Day x Dominance  x Stimulus Position x Response Type | 0.07 | 2,109 | .93 | .001 | 1.04 | 2,109 | .36 | .019 |

The mean RTs and accuracy, defined as numbers of corrected trials, (and relative SD) in the vertical landmark task are reported in Table S4.

**Table S4**. The mean RTs in ms and numbers of trials (and relative SD) of vertical landmark task performed in both testing sessions by each circadian preferences, for hard and easy trials presented in the left- and right-visual field (LVF and RVF respectively).

| RTs | Morning Session | | | | Afternoon Session | | | |
| --- | --- | --- | --- | --- | --- | --- | --- | --- |
|  | **LVF** | | **RVF** | | **LVF** | | **RVF** | |
|  | **Hard** | **Easy** | **Hard** | **Easy** | **Hard** | **Easy** | **Hard** | **Easy** |
| Evening-types | 891  (446) | 836  (501) | 905  (520) | 794  (408) | 902  (433) | 761  (283) | 908  (426) | 796  (359) |
| Intermediate-types | 875  (275) | 765  (233) | 868  (278) | 766  (220) | 887  (294) | 754  (237) | 888  (334) | 752  (222) |
| Morning-types | 920  (315) | 771  (226) | 895  (301) | 795  (301) | 936  (431) | 788  (368) | 934  (445) | 806  (444) |
|  | | | | | | | | |
| Numbers of Corrected Trials | Morning Session | | | | Afternoon Session | | | |
|  | **LVF** | | **RVF** | | **LVF** | | **RVF** | |
|  | **Hard** | **Easy** | **Hard** | **Easy** | **Hard** | **Easy** | **Hard** | **Easy** |
| Evening-types | 9.85  (0.37) | 9.80  (0.41) | 9.70  (0.57) | 9.85  (0.37) | 9.80  (0.41) | 9.85  (0.37) | 9.85  (0.37) | 9.85  (0.37) |
| Intermediate-types | 9.69  (0.53) | 9.79  (0.54) | 9.69  (0.50) | 9.76  (0.43) | 9.61  (0.58) | 9.72  (0.52) | 9.61  (0.65) | 9.76  (0.55) |
| Morning-types | 9.85  (0.37) | 9.81  (0.40) | 9.73  (0.45) | 9.85  (0.37) | 9.69  (0.62) | 9.88  (0.33) | 9.35  (0.63) | 9.85  (0.37) |

In the Supplementary Table 5 (S5), the statistical results of the mixed ANCOVA on mean RTs and accuracy for the landmark task are reported.

**Table S5.** The F value, the degrees of freedom (Df), p value and partial eta-squared (ƞ^2^_p_) are reported for main effects and all interactions. In bold the statistically significant results.

|  | RTs | | | | Accuracy | | | |
| --- | --- | --- | --- | --- | --- | --- | --- | --- |
|  | *F* | Df | *p* | *ƞ^2^_p_* | *F* | Df | *p* | *ƞ^2^_p_* |
| Circadian Preference | 0.27 | 2,109 | .77 | .005 | 2.06 | 2,109 | .13 | .04 |
| Time-of-Day | 0.44 | 1,109 | .51 | .004 | 1.41 | 1,109 | .24 | .013 |
| Task Difficulty | **129.24** | **1,109** | **.00001** | **.54** | **6.24** | **1,109** | **.014** | **.054** |
| Stimulus Position | 2.20 | 1,109 | .14 | .02 | 3.35 | 1,109 | .07 | .03 |
| Circadian Preference x Time-of-Day | 0.002 | 2,109 | .99 | .0001 | 0.70 | 2,109 | .50 | .013 |
| Circadian Preference x Task Difficulty | 0.34 | 2,109 | .72 | .006 | 2.06 | 2,109 | .13 | .04 |
| Circadian Preference x Stimulus Position | 0.37 | 2,109 | .69 | .007 | 0.75 | 2,109 | .48 | .014 |
| Time-of-Day x Task Difficulty | **6.01** | **1,109** | **.016** | **.05** | 0.56 | 1,109 | .46 | .005 |
| Time-of-Day x Stimulus Position | **4.11** | **1,109** | **.045** | **.04** | 0.05 | 1,109 | .83 | .0001 |
| Task Difficulty x Stimulus Position | 1.35 | 1,109 | .25 | .01 | 2.71 | 1,109 | .10 | .024 |
| Circadian Preference x Time-of-Day x Task Difficulty | 0.62 | 2,109 | .54 | .01 | 2.51 | 2,109 | .09 | .04 |
| Circadian Preference x Time-of-Day x Stimulus Position | 1.21 | 2,109 | .30 | .02 | 0.65 | 2,109 | .52 | .012 |
| Circadian Preference x Task Difficulty x Stimulus Position | 1.81 | 2,109 | .17 | .03 | 1.15 | 2,109 | .32 | .021 |
| Time-of-Day x Task Difficulty x Stimulus Position | 0.019 | 1,109 | .89 | .00001 | 0.02 | 1,109 | .90 | .0001 |
| Circadian Preference x Time-of-Day  X Task Difficulty x Stimulus Position | 0.99 | 2,109 | .38 | .02 | 0.58 | 2,109 | .56 | .01 |
